# Inhibition of miR-508-5p Promotes Osteogenic–Angiogenic Coupling to Enhance Bone Formation

**DOI:** 10.3390/ijms27188390

**Published:** 2026-09-20

**Authors:** Jing Guo, Xiangying Ouyang, Jianru Liu, Wenyi Liu, Ruifang Lu

**Affiliations:** 1 Department of Periodontology, Peking University School and Hospital of Stomatology, Beijing 100081, China; 1710303114@pku.edu.cn (J.G.); lensang@163.com (J.L.); liuwenyi0000@126.com (W.L.); 2 National Center for Stomatology, National Clinical Research Center for Oral Diseases, Beijing 100081, China; 3 National Engineering Research Center of Oral Biomaterials and Digital Medical Devices, Beijing 100081, China

**Keywords:** miR-508-5p, periodontal ligament stem cells, angiogenesis, osteogenic–angiogenic coupling, bone regeneration

## Abstract

Periodontal bone regeneration depends on the coordinated interplay between osteogenesis and angiogenesis; however, the molecular regulation underlying osteogenic–angiogenic coupling remains incompletely understood. Despite previous evidence that miR-508-5p downregulation enhances the osteogenic differentiation of human periodontal ligament stem cells (hPDLSCs) in vitro, its role in regulating angiogenesis and bone formation in vivo remains unclear. In the present study, miR-508-5p was modulated using synthetic inhibitor and mimic oligonucleotides, and its effects on osteogenesis, angiogenesis, and osteogenic–angiogenic coupling were investigated by the use of alkaline phosphatase and alizarin red S staining, conditioned-medium assays, a chicken chorioallantoic membrane assay, and a nude mouse ectopic bone formation model. The responses to miR-508-5p inhibition differed by cell type: osteogenic differentiation increased in hPDLSCs, while human umbilical vein endothelial cells (HUVECs) showed greater proliferative, migratory, and tube-forming activity. Conditioned medium experiments further revealed that miR-508-5p inhibition enhanced the reciprocal paracrine effects between hPDLSCs and HUVECs, thereby promoting both osteogenic and angiogenic responses. In vivo, miR-508-5p inhibition enhanced bone-like tissue formation and collagen deposition, accompanied by the elevated expression of collagen type I alpha 1 chain, bone morphogenetic protein 2, and the angiogenic marker CD31. Taken together, the findings support a potential regulatory role for miR-508-5p in osteogenic–angiogenic coupling and indicate that this microRNA may warrant consideration as a candidate target in therapeutic strategies for periodontal bone regeneration.

## 1. Introduction

Periodontitis is a substantial component of the global tooth-loss burden and a chronic inflammatory disorder in which dental plaque biofilms initiate the disease process [1]. Alveolar bone destruction is characteristic of periodontitis; it also represents a major obstacle for periodontal regeneration [2]. Successful periodontal regeneration requires effective bone formation [3].

Bone formation is a highly coordinated process involving complex interactions among multiple cell types [4]. A close coordination between osteogenesis and angiogenesis occurs during bone formation, while this osteogenic–angiogenic coupling is central to maintaining bone homeostasis and facilitating bone regeneration [5,6,7,8]. Endothelial cells provide essential signals to support osteogenic differentiation, while osteogenic cells can regulate vascular formation through paracrine mechanisms [9,10]. The combined osteogenic capacity and ability to regulate the angiogenesis of periodontal ligament stem cells (PDLSCs) support their potential utility in periodontal regeneration, as indicated by recent evidence.

Approaches using stem cells as the basis for therapies have recently drawn increasing interest in periodontal regeneration [11]. For periodontal tissue regeneration, human periodontal ligament stem cells (hPDLSCs) offer promise as a seed-cell source [12]. However, the biological functions of hPDLSCs are influenced by the inflammatory microenvironment associated with periodontitis [13]. Prior evidence indicates that inflammatory stimulation weakens osteogenic differentiation in hPDLSCs and, consequently, limits their contribution to bone regeneration [14,15]. In addition to compromised osteogenesis, inflammation-induced endothelial cell dysfunction represents another critical factor affecting bone formation. Inflammatory stimulation has also been reported to disrupt endothelial cell migration and tube formation, ultimately reducing the angiogenic capacity [16]. Given that angiogenesis is essential for supporting osteogenesis during bone formation, inflammation-associated alterations in both osteogenic differentiation and angiogenesis may lead to the disruption of osteogenic–angiogenic coupling and defective periodontal bone formation. Therefore, identifying novel molecular targets that simultaneously enhance the osteogenic potential of hPDLSCs and modulate endothelial angiogenic function is essential to devising clinically effective approaches for promoting the regeneration of periodontal bone.

The functional properties of hPDLSCs can be enhanced by various strategies, including pharmacological stimulation, immunomodulation, and epigenetic regulation [17]. Epigenetic regulation has also emerged as an attractive strategy because it can regulate gene expression while leaving the underlying DNA sequence unchanged. microRNAs are critical to periodontal tissue homeostasis and osteogenic lineage differentiation in mesenchymal stem cells (MSCs) and have been implicated in bone regeneration [18,19]. In our previous in vitro work, we found that the inhibition of miR-508-5p enhanced the osteogenic response of hPDLSCs by directly targeting SRY-box transcription factor 11 (*SOX11*), which was confirmed by luciferase reporter assays and rescue experiments [20]. However, whether miR-508-5p regulates in vivo bone formation and whether it affects endothelial angiogenic function and osteogenic–angiogenic coupling remains incompletely understood.

This study therefore addressed whether the modulation of miR-508-5p alters bone formation through changes in osteogenic–angiogenic coupling. The present study may uncover a potential molecular target and a novel strategy for periodontal bone regeneration.

## 2. Results

### 2.1. Characterization of hPDLSCs

**Figure 1:**
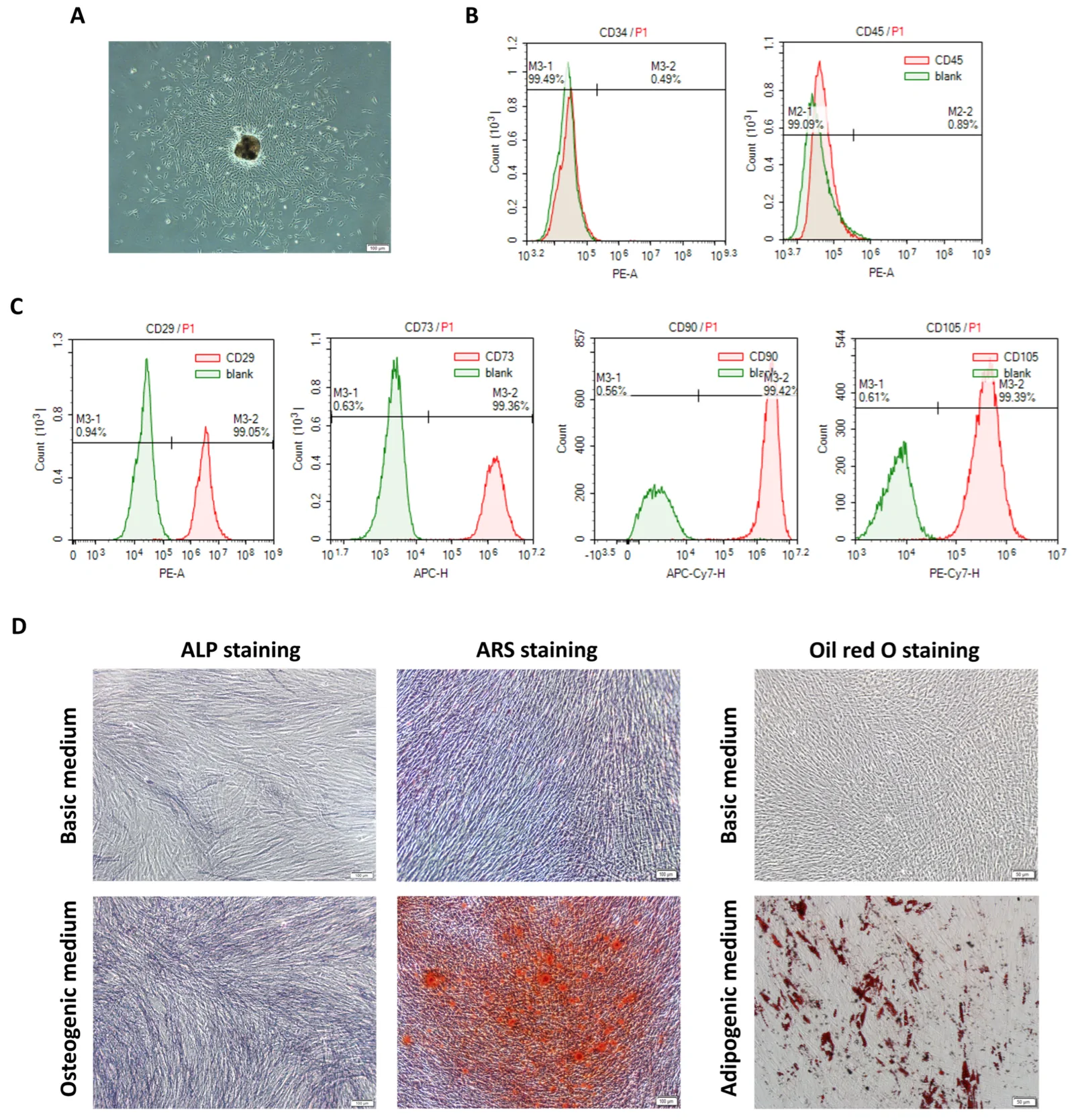
Phenotypic features supporting identification of hPDLSCs in culture. (**A**) Representative morphology of hPDLSCs under culture conditions (scale bar: 100 μm). (**B**) Flow-cytometric assessment indicating lack of CD34 and CD45 expression. (**C**) Positive flow-cytometric signals for CD73, CD29, CD105, and CD90. (**D**) During osteogenic induction, day-7 ALP staining and day-21 ARS staining were implemented (scale bar: 100 μm); adipogenesis was confirmed by day-21 ORO staining (scale bar: 50 μm). hPDLSCs, human periodontal ligament stem cells; ALP, alkaline phosphatase; ARS, alizarin red S; ORO, Oil red O.

The isolated hPDLSCs were identified as MSCs using the profile of specific surface markers together with their multilineage differentiation capability (Figure 1A). Flow cytometry showed a contrast in the marker profile of hPDLSCs: there was a strong expression of the MSC-associated markers CD29, CD73, CD90, and CD105, whereas hematopoietic markers CD34 and CD45 were negatively expressed (Figure 1B,C). Furthermore, hPDLSCs cultured under osteogenic induction conditions exhibited increased alkaline phosphatase (ALP) activity, as demonstrated by ALP staining, showed apparent mineralized nodule formation, as confirmed by alizarin red S (ARS) staining. In contrast, adipogenic induction resulted in the formation of abundant lipid droplets, as evidenced by Oil red O (ORO) staining (Figure 1D). These results show that the isolated hPDLSCs possess the defining characteristics of MSCs.

### 2.2. miR-508-5p Inhibition Enhances Osteogenic Differentiation, Proliferation, and Migration of hPDLSCs

**Figure 2:**
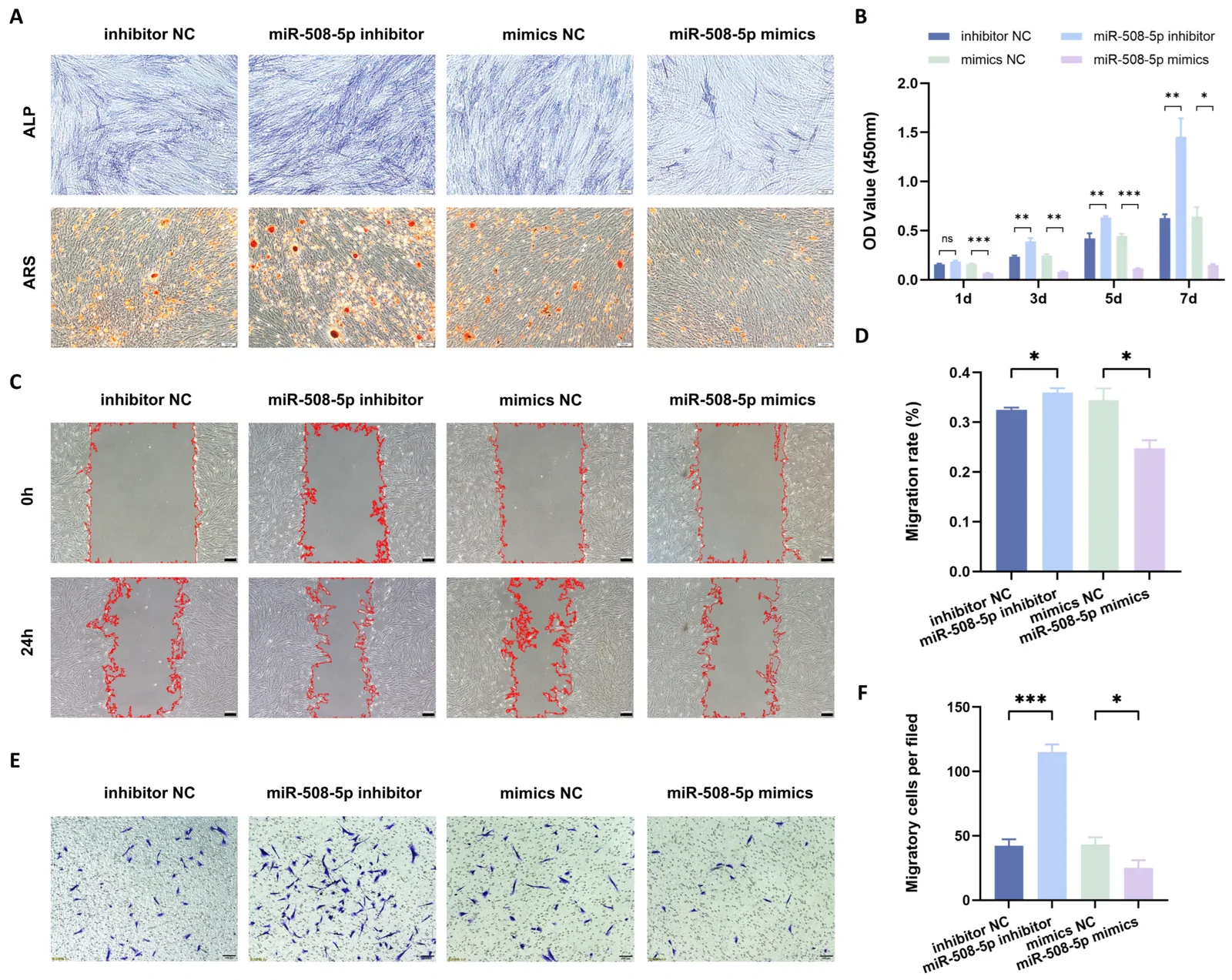
miR-508-5p inhibition enhances osteogenic differentiation, proliferation, and migration of hPDLSCs. (**A**) During osteogenic induction, day-7 ALP staining and day-21 ARS staining were implemented (scale bar: 100 μm). (**B**) CCK-8-based measurement of hPDLSC proliferation across days 1, 3, 5, and 7. (**C**) Wound healing assessment of hPDLSC migration (scale bar: 200 μm) with (**D**) associated quantification. (**E**) Transwell assay evaluating hPDLSC migration (scale bar: 100 μm) and (**F**) corresponding quantification. * *p* < 0.05, ** *p* < 0.01, *** *p* < 0.001. ns, not significant. CCK8, Cell Counting Kit-8.

Transfected hPDLSCs were cultured in osteogenic medium (OM) for 7 or 21 days as the experimental basis for assessing how miR-508-5p affects osteogenic differentiation. ALP and ARS staining demonstrated that the inhibition of miR-508-5p enhanced ALP activity and mineral deposition, whereas miR-508-5p overexpression through mimic transfection reduced the ALP activity and mineralization levels (Figure 2A). Our current pattern is consistent with earlier evidence [20] that miR-508-5p restrains osteogenic differentiation in hPDLSCs.

Changes in hPDLSC proliferation associated with miR-508-5p modulation were examined using the Cell Counting Kit-8 (CCK-8) assay. Except for day 1, cells receiving the miR-508-5p inhibitor exhibited significantly increased absorbance values relative to the miR-508-5p inhibitor negative control (inhibitor NC) group across days 3, 5, and 7. Relative to miR-508-5p mimic negative control (mimic NC), miR-508-5p mimic-transfected cells showed lower absorbance values; overall, the proliferation results indicate that miR-508-5p inhibition favors hPDLSC proliferation, whereas overexpression suppresses it (Figure 2B).

miR-508-5p’s influence on hPDLSC migration was assessed with wound healing and Transwell assays. Both migration readouts increased after miR-508-5p inhibitor transfection relative to the inhibitor NC: wound closure was faster, and more cells traversed the Transwell membrane. Conversely, miR-508-5p mimic transfection resulted in delayed wound closure and fewer migrated cells compared to the mimic NC group (Figure 2C–F). Collectively, the combined findings demonstrate that miR-508-5p inhibition promotes osteogenic differentiation while enhancing the proliferative and migratory capacities of hPDLSCs.

### 2.3. miR-508-5p Inhibition Promotes HUVECs Proliferation, Migration, and Angiogenesis

**Figure 3:**
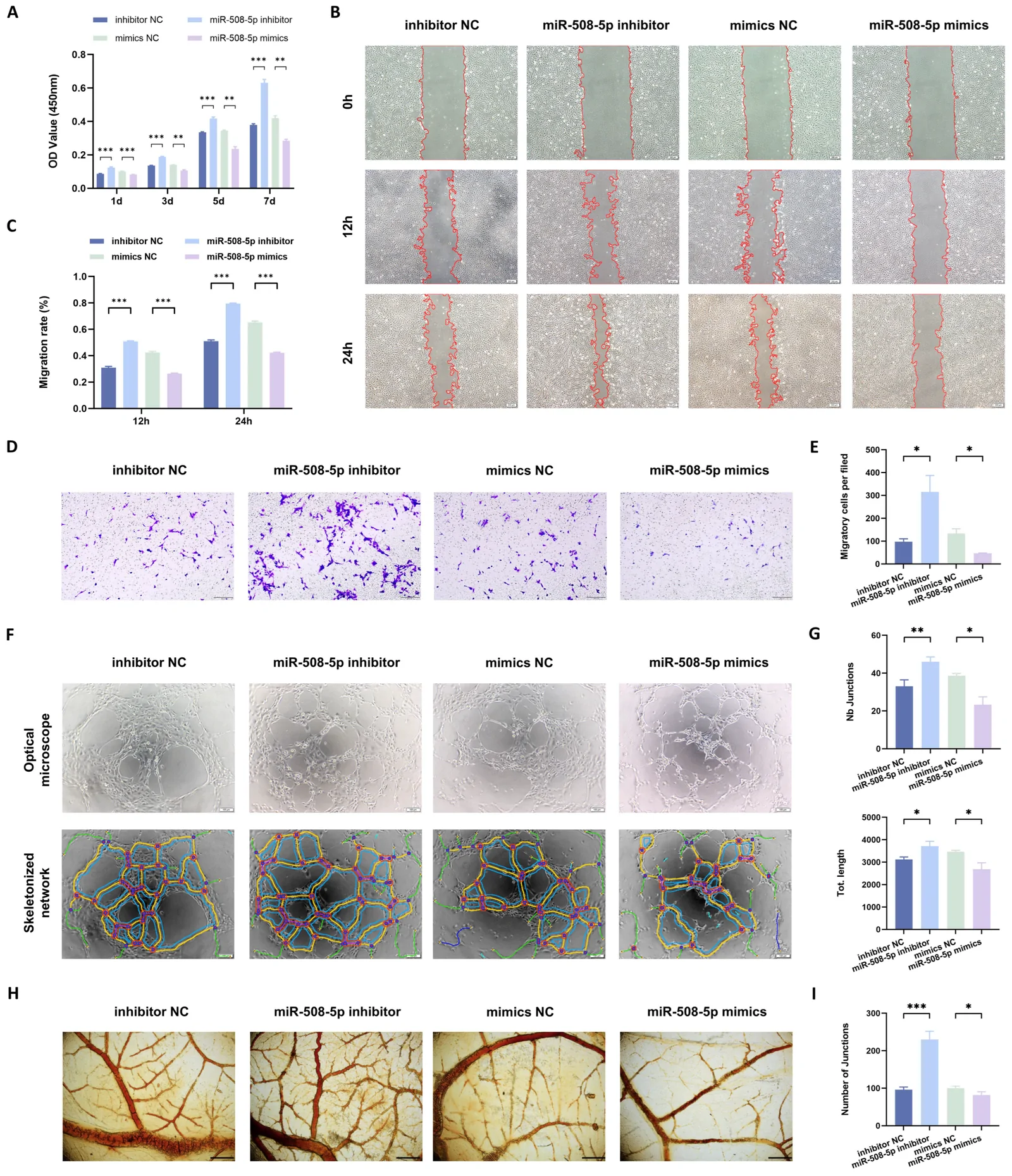
miR-508-5p inhibition promotes HUVECs proliferation, migration, and angiogenesis. (**A**) HUVECs proliferation measured by CCK-8 across days 1, 3, 5, and 7. (**B**) Wound healing assessment to evaluate HUVECs migration (scale bar: 200 μm) with (**C**) corresponding quantitative analysis. (**D**) Migration of HUVECs in the Transwell assay (scale bar: 200 μm); (**E**) quantification of the panel-D migration readout. (**F**) Matrigel tube-formation assay conducted in vitro (scale bar: 100 μm); (**G**) quantification of junction numbers and total tube length from panel F. (**H**) In vivo CAM assay to evaluate angiogenesis (scale bar: 500 μm) and (**I**) associated quantification of junction numbers. * *p* < 0.05, ** *p* < 0.01, *** *p* < 0.001. HUVECs, human umbilical vein endothelial cells; CAM, chicken embryo chorioallantoic membrane.

CCK-8 assays showed that miR-508-5p inhibition enhanced human umbilical vein endothelial cells (HUVECs) proliferation, whereas miR-508-5p overexpression exerted the opposite effect compared with the corresponding controls (Figure 3A).

Faster wound closure was observed after miR-508-5p inhibition relative to inhibitor NC, reaching approximately 50% at 12 h and 80% at 24 h. This wound healing assay result formed one component of the migration assessment in HUVECs, with the Transwell assay providing the complementary migration readout. In contrast, miR-508-5p mimic transfection delayed the wound healing process, with only 42% wound closure observed at 24 h relative to the mimic NC group (Figure 3B,C). Consistently, Transwell assays showed a similar trend, indicating that miR-508-5p inhibition enhanced both the horizontal and vertical migration capacities of HUVECs, whereas miR-508-5p overexpression impaired their migratory ability (Figure 3D,E).

The angiogenic performance in HUVECs was examined in two settings: an in vitro Matrigel tube formation assay and an in vivo chicken embryo chorioallantoic membrane (CAM) assay. In the Matrigel tube formation assay, the miR-508-5p inhibitor group exhibited 46 junctions with a total tube length of 3711.3 arbitrary units (a.u.), representing approximately 1.4-fold and 1.2-fold increases relative to the inhibitor NC group, respectively. Conversely, the group treated with miR-508-5p mimics displayed 23 junctions and a total tube length of 2678 a.u., corresponding to approximately 60% and 77% of those observed in the mimic NC group, respectively (Figure 3F,G). In the CAM assay, the inhibition of miR-508-5p enhanced angiogenesis, with the inhibitor group showing 230 branch points, which was approximately 2.4-fold higher than the inhibitor NC group. In contrast, the miR-508-5p mimic group exhibited 80 branch points, corresponding to approximately 0.8-fold of the mimic NC group (Figure 3H,I). Collectively, these findings demonstrate that the inhibition of miR-508-5p enhances HUVECs proliferation, migration, and angiogenic potential.

### 2.4. miR-508-5p Inhibition Enhances Conditioned-Medium-Mediated Osteogenic–Angiogenic Coupling

**Figure 4:**
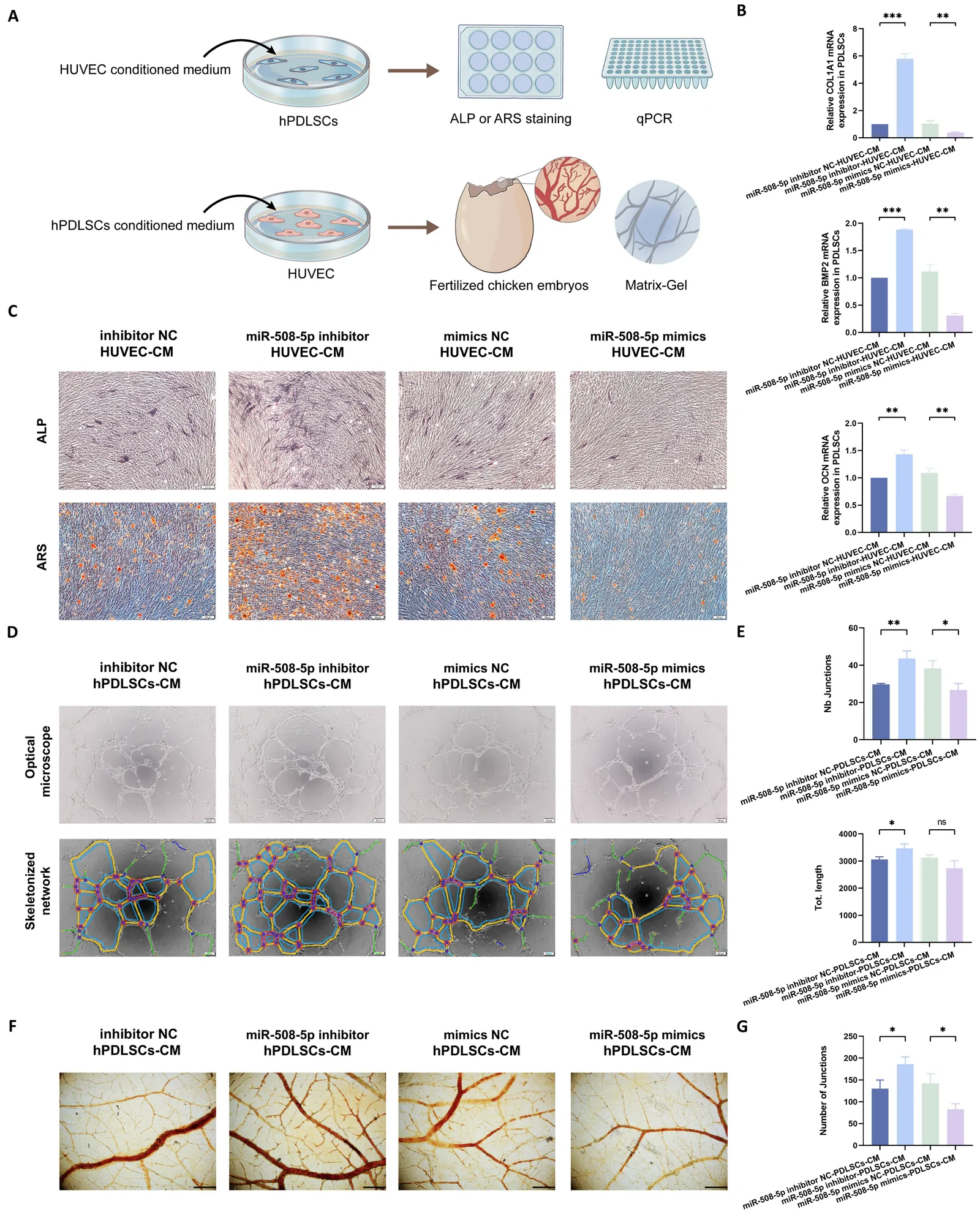
miR-508-5p inhibition enhances conditioned-medium-mediated osteogenic–angiogenic coupling. (**A**) A schematic diagram illustrating the experimental design using conditioned medium derived from transfected cells for evaluate osteogenesis and angiogenesis. (**B**) Relative mRNA expression levels of osteogenic genes (*COL1A1*, *BMP2*, and *OCN*) in CM-treated hPDLSCs. (**C**) Day-7 ALP staining and day-21 ARS staining were implemented during osteogenic induction (scale bar: 100 μm). (**D**) In vitro Matrigel tube formation assessment of HUVECs and (**E**) associated quantification of junction numbers and total tube length (scale bar: 200 μm). (**F**) In vivo angiogenesis assessment using CAM (scale bar: 500 μm) and (**G**) junction number quantification corresponding to panel F. * *p* < 0.05, ** *p* < 0.01, *** *p* < 0.001. ns, not significant. *COL1A1*, collagen type I alpha 1 chain; *BMP2*, bone morphogenetic protein 2; *OCN*, osteocalcin.

To investigate whether miR-508-5p regulation in hPDLSCs and HUVECs affects the interaction between osteogenesis and angiogenesis, the conditioned medium (CM) was collected from transfected hPDLSCs or HUVECs and used to treat the other cell type for subsequent functional assays (Figure 4A).

The CM from HUVECs transfected with an miR-508-5p inhibitor or mimic was applied to hPDLSCs, after which osteogenic induction was performed to assess the osteogenic response of the recipient hPDLSCs. In hPDLSCs, quantitative reverse-transcription polymerase-chain reaction (qRT-PCR) detected a higher collagen type I alpha 1 chain (*COL1A1*), bone morphogenetic protein 2 (*BMP2*), and osteocalcin (*OCN*) expression after treatment with CM from miR-508-5p inhibitor-transfected HUVECs (Figure 4B). Consistently, ALP and ARS staining demonstrated enhanced ALP activity together with greater mineralized nodule formation in hPDLSCs treated with the inhibitor-derived CM. In contrast, the CM from miR-508-5p mimic-transfected HUVECs reduced the osteogenic gene expression, ALP activity, and mineral deposition (Figure 4C). The findings support the greater osteogenic differentiation of hPDLSCs in response to the CM from HUVECs subjected to miR-508-5p inhibition.

For the reciprocal experiment, the CM from transfected hPDLSCs was collected and then applied to HUVECs, whose angiogenic response was evaluated by Matrigel tube formation and CAM assays. Compared with the inhibitor NC group, the CM derived from miR-508-5p inhibitor-transfected hPDLSCs enhanced the tube formation capacity, characterized by increased branch points and total tube length. In contrast, CM from miR-508-5p mimic-transfected hPDLSCs exhibited reduced angiogenic activity compared with mimic NC-derived CM (Figure 4D,E). Similarly, CAM assays further demonstrated that the inhibitor-derived CM promoted in vivo vascular formation and resulted in an increased number of branch points (Figure 4F,G). These results suggest that miR-508-5p inhibition enhances the ability of the hPDLSC-derived CM to stimulate angiogenic activity in HUVECs.

Collectively, these findings demonstrate that hPDLSCs and HUVECs exhibit the reciprocal regulation of osteogenic differentiation and angiogenic activity through conditioned-medium-mediated interactions, and the inhibition of miR-508-5p strengthens this osteogenic–angiogenic coupling.

### 2.5. miR-508-5p Inhibition Promotes Vascularized Bone Formation In Vivo

**Figure 5:**
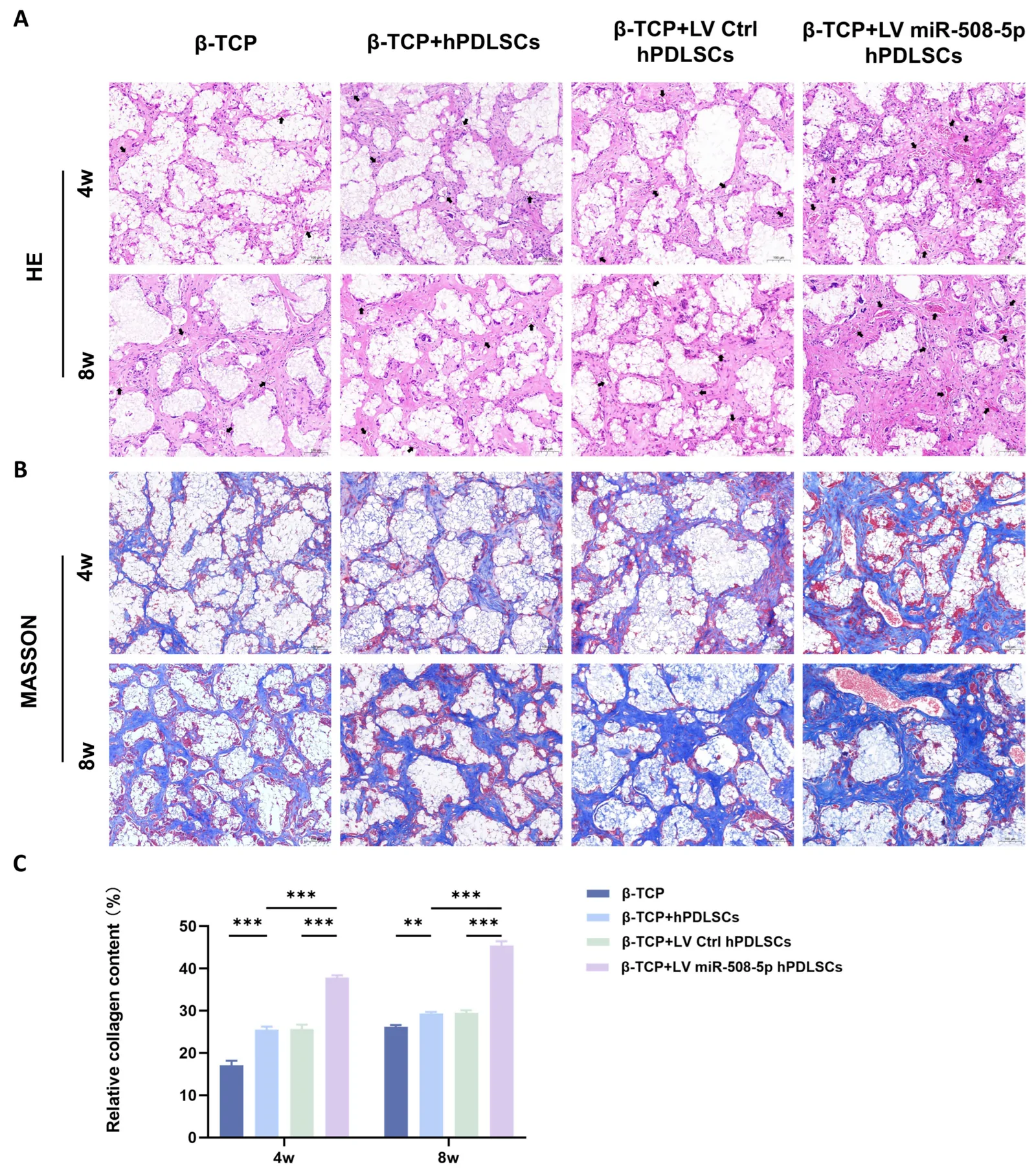
Assessment of in vivo bone formation and vascularization in a subcutaneous model at 4 and 8 weeks. (**A**) HE staining images (scale bar: 100 μm) with arrows indicating vascular structures. (**B**) Masson staining images (scale bar: 100 μm). (**C**) Quantitative analysis of collagen deposition. ** *p* < 0.01, *** *p* < 0.001. HE, hematoxylin and eosin.

A subcutaneous ectopic bone formation model was constructed from β-tricalcium phosphate (β-TCP) scaffolds carrying hPDLSCs from the different treatment groups to examine the in vivo effect of miR-508-5p inhibition. A histological evaluation was performed by hematoxylin and eosin (HE) staining and Masson staining (Figure 5).

HE staining revealed the progressive formation of bone-like tissues in all groups over time, accompanied by the appearance of newly formed vascular structures (black arrows) within the tissues. The vascular structures were identified by the presence of densely distributed orange–red “disc-like” structures representing red blood cells, which exhibited central pale staining and no visible nuclei, together with the surrounding lumen-like structures containing these red blood cells. At 4 weeks, residual β-TCP particles occupied most of the scaffold spaces in the β-TCP, β-TCP + hPDLSC, and β-TCP + LV Ctrl (control lentivirus) hPDLSC groups, with only limited newly formed tissues observed. Relative to the β-TCP group, all three hPDLSC-containing groups showed a greater formation of bone-like tissues. Notably, the β-TCP + LV miR-508-5p (lentivirus-mediated miR-508-5p knockdown) hPDLSC group showed more extensive eosinophilic bone-like tissues. At 8 weeks, the further expansion and maturation of bone-like tissues were observed, particularly in the β-TCP + LV miR-508-5p hPDLSC group, where newly formed tissues exhibited more organized structures and were associated with increased vascular structures displaying an enlarged luminal morphology (Figure 5A).

Masson staining was performed to assess the collagen-rich matrix formation. At 4 weeks, the β-TCP, β-TCP + hPDLSC, and β-TCP + LV Ctrl hPDLSC groups displayed relatively weak blue staining with loosely arranged collagen fibers. In contrast, collagen deposition was enhanced in the hPDLSC-containing groups, with the β-TCP + LV miR-508-5p hPDLSC group showing a broader area of blue staining and more abundant collagen matrix formation. Newly formed vascular structures were also observed within the collagenous matrix, characterized by vascular lumens of different sizes containing erythrocytes. At 8 weeks, collagen fibers became denser and more organized in all groups, while vascular structures became more evident. Compared with the other groups, the β-TCP + LV miR-508-5p hPDLSC group exhibited more extensive collagen deposition, better-organized collagen structures, and more prominent vascular features (Figure 5B,C).

The in vivo bone-forming effect of miR-508-5p inhibition was characterized by enhanced collagen deposition and vascular structure development.

### 2.6. miR-508-5p Inhibition Promotes Osteogenic and Angiogenic Marker Expression During In Vivo Bone Formation

**Figure 6:**
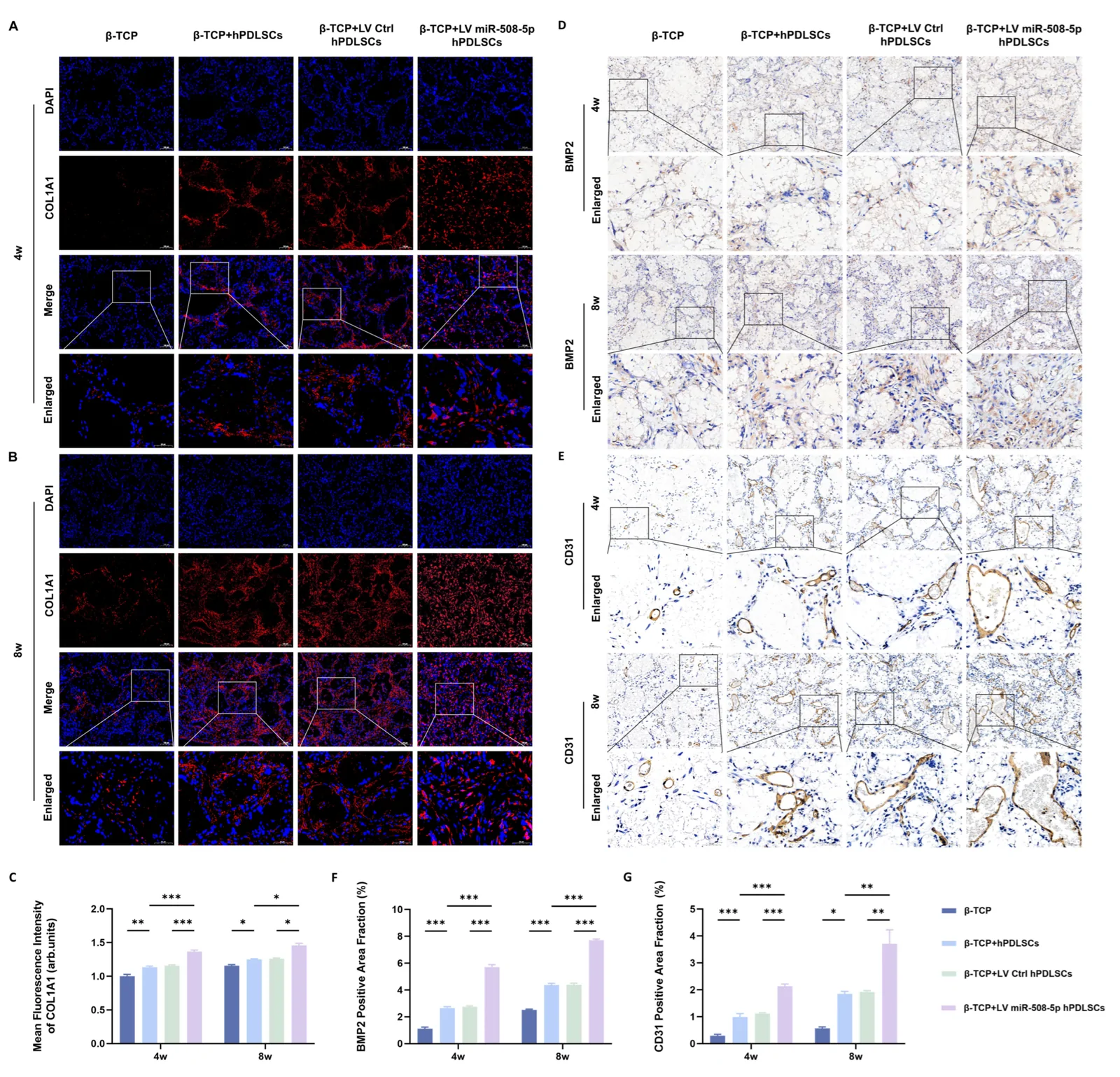
Assessment of osteogenic and angiogenic marker expression in a subcutaneous heterotopic bone formation model at 4 and 8 weeks. (**A**,**B**) Representative immunofluorescence staining images depicting COL1A1 expression (scale bar: 100 μm). (**C**) Quantitative assessment of COL1A1 expression. (**D**) Immunohistochemical staining of BMP2 (scale bar: 100 μm). (**E**) Immunohistochemical staining of CD31 (scale bar: 100 μm). (**F**) Quantitative assessment of BMP2 expression. (**G**) Quantitative assessment of CD31 expression. * *p* < 0.05, ** *p* < 0.01, *** *p* < 0.001.

To assess osteogenic differentiation and vascularization during in vivo bone formation under miR-508-5p inhibition, osteogenic markers (COL1A1 and BMP2) and the angiogenic marker CD31 were evaluated by immunofluorescence and immunohistochemical staining (Figure 6).

COL1A1, a major component of type I collagen, was examined as an indicator of newly formed bone tissue. Immunofluorescence staining showed blue fluorescence in nuclei and red fluorescence representing COL1A1-positive signals. At both 4 and 8 weeks, all hPDLSC-containing groups exhibited stronger COL1A1 fluorescence signals compared with the β-TCP group. Among these groups, the β-TCP + LV miR-508-5p hPDLSC group displayed the highest COL1A1 fluorescence intensity, indicating increased collagen production and osteogenic activity (Figure 6A,B).

Consistently, the immunohistochemical staining of BMP2 showed brownish-yellow positive signals. At both 4 and 8 weeks, the β-TCP + LV miR-508-5p hPDLSC group exhibited stronger BMP2-positive staining compared with the other groups. The representative images and quantitative analysis indicated the increased expression of this osteogenic marker (Figure 6C,E).

For the angiogenic evaluation, CD31 immunohistochemical staining was performed, with CD31-positive vascular structures represented by brownish-yellow signals. At both time points, the β-TCP group without hPDLSCs showed the lowest number of CD31-positive structures. Compared with the other groups, the β-TCP + LV miR-508-5p hPDLSC group demonstrated a stronger CD31 expression. In the quantitative comparison, the greatest CD31-positive vessel count was recorded for this group (Figure 6D,E).

Collectively, these findings demonstrate that the inhibition of miR-508-5p upregulates osteogenic and angiogenic marker expression, supporting its role in promoting in vivo bone formation through strengthened osteogenic–angiogenic coupling.

## 3. Discussion

Chronic and persistent inflammation represents a major obstacle to effective periodontal bone regeneration, as it compromises both the stem cell osteogenic differentiation capacity and endothelial cell angiogenic function [16,21,22]. The critical contribution of bone vasculature to bone formation and homeostasis has been increasingly recognized [23,24]. The present work extended our earlier in vitro finding [20]—that miR-508-5p downregulation enhances osteogenic differentiation in hPDLSCs—by examining its relevance to bone formation. To our knowledge, this study provides initial evidence that the inhibition of miR-508-5p enhances the osteogenic–angiogenic coupling between hPDLSCs and HUVECs, thereby promoting bone formation in vivo.

The first part of this study evaluated the biological properties of hPDLSCs following miR-508-5p inhibition. As promising seed cells for periodontal regeneration, hPDLSCs show a self-renewal capacity while also displaying multilineage differentiation potential, including differentiation toward osteoblasts, cementoblasts, and periodontal ligament (PDL) cells [25,26]. The evidence of enhanced osteogenic differentiation came from increased ALP-positive staining and mineralized nodule formation under induction conditions after miR-508-5p inhibition. Moreover, miR-508-5p inhibition increased proliferation and migration in hPDLSCs, a pattern suggesting broader involvement in their biological functions during bone formation.

The essential contribution of angiogenesis to bone formation and osteogenic–angiogenic coupling [27] provided the rationale for examining how miR-508-5p modulation affects the proliferative activity, migration, and angiogenic capacity in HUVECs. Angiogenesis studies commonly use HUVECs as an in vitro model, and HUVECs also play important roles in vascular formation [28]. At the phenotypic level, the present study demonstrated that endothelial responses varied according to the direction of miR-508-5p modulation: inhibition enhanced migration and tube formation, whereas overexpression produced the opposite effects. Consistent with our findings, previous studies have also suggested a role for miR-508-5p in vascular regulation. Liu et al. [29] reported that a higher miR-508-5p expression in endothelial progenitor cells was associated with an impaired angiogenic capacity and suggested that this effect may involve angiogenesis-related targets such as glucose transporter type 1 (*GLUT1*). GLUT1 is a major facilitative glucose transporter that mediates the cellular glucose uptake and plays an important role in glycolytic metabolism. Regarding the potential molecular mechanisms underlying the angiogenic effects of miR-508-5p, the present study further demonstrated, using a dual-luciferase reporter assay, that miR-508-5p directly targets the *GLUT1* 3′-UTR, providing experimental evidence for *GLUT1* as a downstream target of miR-508-5p. Accumulating evidence supports an important role for GLUT1-mediated glucose metabolism in angiogenesis. Metabolic reprogramming through the co-delivery of *GLUT1* and hexokinase 2 mRNAs has been shown to enhance glycolytic activity, cell proliferation, extracellular matrix synthesis, and paracrine-mediated angiogenesis, ultimately promoting vascularized dental pulp tissue regeneration [30]. These findings, together with our direct targeting evidence, suggest that miR-508-5p may influence angiogenic activity through the regulation of GLUT1-dependent glucose metabolism and glycolytic activity. Importantly, GLUT1 is also closely associated with osteogenesis. Previous work demonstrated that GLUT1-mediated glucose uptake promotes osteoblast differentiation and bone formation by supporting Runt-related transcription factor 2 (*RUNX2*) accumulation and collagen synthesis, while *RUNX2* reciprocally enhances *GLUT1* expression, thereby forming a feed-forward regulatory loop between glucose metabolism and osteogenesis [31]. Overall, these findings suggest that the miR-508-5p/GLUT1-mediated metabolic pathway may represent a potential molecular link between osteogenic and angiogenic processes and may contribute to the osteogenic–angiogenic coupling effects of miR-508-5p.

Coordinated vascular formation occurs alongside the osteogenic differentiation of MSCs during bone formation [32,33]. hPDLSCs possess osteogenic potential and are closely associated with vascular formation. Previous studies have demonstrated that PDLSCs can regulate endothelial angiogenesis through paracrine mechanisms, including the exosome-mediated transfer of angiogenic factors [34,35]. Paracrine signaling represents an important mode of communication between cells, which is achieved through the release of soluble factors, protein complexes, and packaged mediators [36]. In the present study, conditioned medium derived from miR-508-5p-modulated hPDLSCs applied to HUVECs resulted in increased junction numbers and total tube length, indicating an enhanced angiogenic capacity and further supporting the role of PDLSCs in regulating endothelial angiogenesis through paracrine effects. Together with the findings that the direct modulation of miR-508-5p in HUVECs altered their intrinsic angiogenic properties, these results suggest that the effects of miR-508-5p regulation on endothelial function may involve both direct and indirect mechanisms. Conversely, the conditioned medium derived from miR-508-5p-modulated HUVECs applied to hPDLSCs produced an increase in the osteogenic-gene expression (*COL1A1*, *BMP2*, and *OCN*), enhanced ALP activity, and greater formation of mineralized nodules. These findings suggest that endothelial-derived paracrine signals can reciprocally enhance the osteogenic potential of hPDLSCs. This part of the study provides evidence that the inhibition of miR-508-5p enhances the bidirectional, paracrine-mediated communication between hPDLSCs and HUVECs, highlighting the critical role of miR-508-5p in this complex cellular crosstalk.

**Figure 7:**
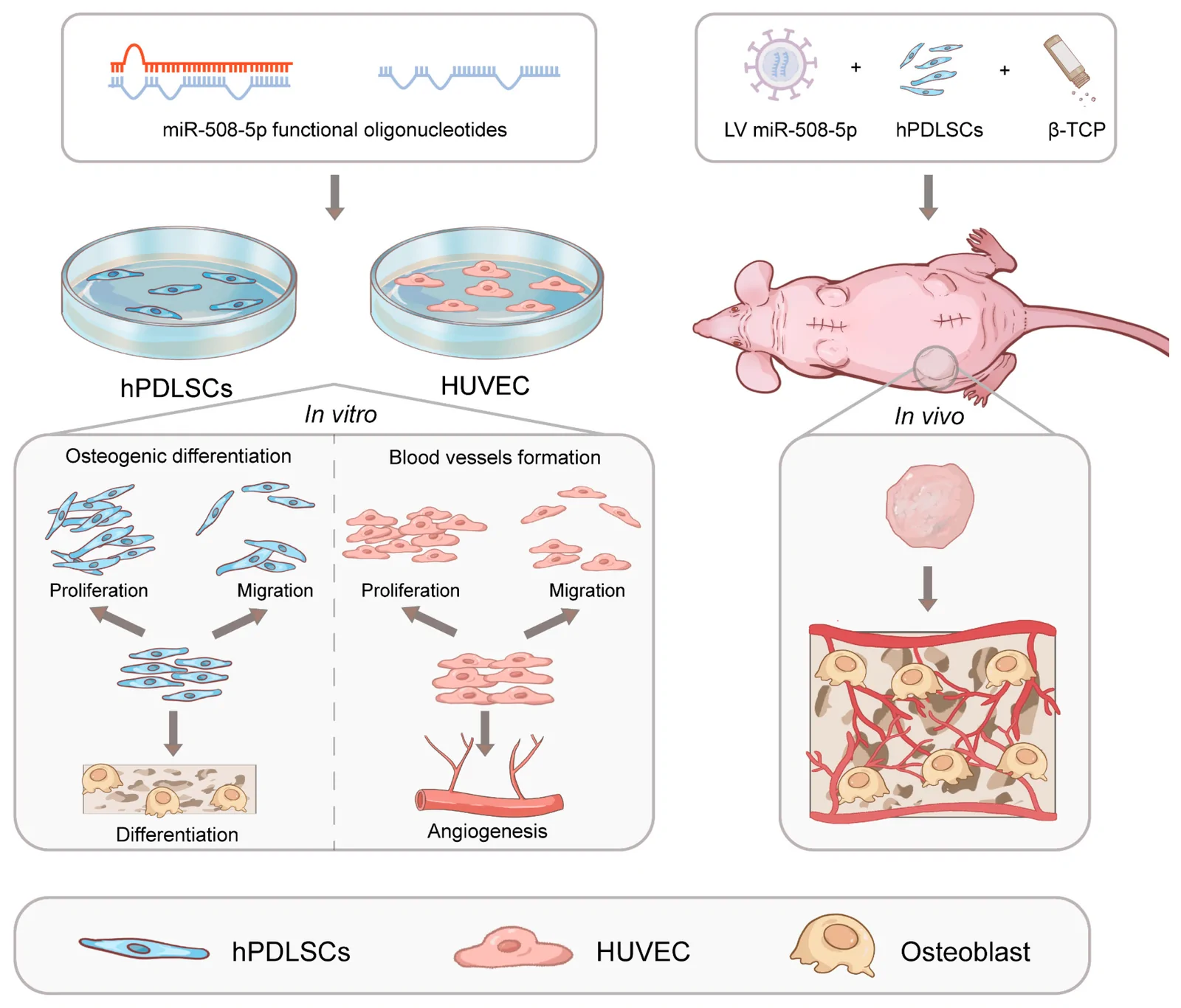
Schematic illustration of miR-508-5p inhibition-mediated osteogenic–angiogenic coupling and bone formation.

Bone formation is a dynamic process tightly coupled with angiogenesis, as bone tissue has an extensive vascular supply [37,38]. The ectopic subcutaneous model further provided the in vivo validation of these findings. In that setting, miR-508-5p inhibition significantly accelerated collagen accumulation and increased both new bone formation and associated vascular structures. The marker profile in newly formed tissues provided complementary evidence: miR-508-5p inhibition increased the expression of the osteogenic markers COL1A1 and BMP2 together with the angiogenic marker CD31, supporting the involvement of this miRNA in osteogenic–angiogenic coupling and bone formation in vivo (Figure 7).

This study has several limitations that should be acknowledged. Although the present study demonstrated that the inhibition of miR-508-5p enhanced the osteogenic differentiation of hPDLSCs, osteogenic outcomes were assessed using a limited number of readouts, and the signaling pathways underlying miR-508-5p-mediated osteogenic regulation remain incompletely elucidated. Moreover, the corresponding negative controls were included to minimize potential nonspecific effects associated with oligonucleotide transfection and lentiviral transduction, thereby supporting the reliability of the observed effects. The inclusion of blank controls alongside the negative-control groups in future studies could provide an additional reference for evaluating potential nonspecific effects. Furthermore, although the consistent findings obtained using different approaches to modulate miR-508-5p support the robustness of the observed regulatory effects, additional experiments, such as rescue assays or a more systematic evaluation of potential off-target effects, could further strengthen the evidence for the specificity of miR-508-5p-mediated regulation. In addition, while enhanced bone formation and vascularization were demonstrated through histological analysis and the evaluation of key markers, the subcutaneous ectopic bone formation model used in this study does not fully recapitulate the complex microenvironment associated with periodontal bone destruction. Further work in more clinically relevant settings, including periodontal bone defect models, is justified to corroborate these findings and to examine the translational prospects of miR-508-5p-based strategies for promoting bone formation in vivo. Finally, although the present study identified *GLUT1* as a direct target of miR-508-5p, the downstream metabolic events connecting miR-508-5p/*GLUT1* regulation with osteogenic–angiogenic coupling remain to be further investigated. Future studies are needed to clarify how this regulatory axis influences the metabolic and cellular processes underlying osteogenic–angiogenic coupling.

In summary, this study identifies miR-508-5p as a regulator of the osteogenic differentiation of hPDLSCs and osteogenic–angiogenic coupling. These findings provide new insights into the regenerative potential of hPDLSCs as seed cells and identify the therapeutic promise in targeting miR-508-5p for periodontal vascularized bone regeneration. Further validation in clinically relevant periodontal defect models is warranted to establish the translational potential of miR-508-5p-targeted therapies.

## 4. Materials and Methods

### 4.1. Cell Culture

PDL tissues of healthy premolars extracted from six donors aged 18 to 25 years at Peking University Stomatology Hospital was obtained. The PDL tissue was removed from the root of the teeth. Tissue fragments (1–5 mm) were centrifuged and seeded in inverted flasks (NEST, Wuxi, China). After 12 h of culture, the flasks were returned to the upright position. Following replacement of culture media at 48 h intervals, a limited number of dilutions was performed to passage cell outgrowths. The maintenance medium for hPDLSCs consisted of Alpha Minimum Essential Medium (α-MEM) (Gibco, Grand Island, NY, USA) containing 10% FBS and 1% penicillin–streptomycin. All hPDLSCs used in this study were between passages P3 and P5. The Ethics Committee of Peking University Hospital of Stomatology approved the study (PKUSSIRB-202497033).

The culture medium for HUVECs was Endothelial Cell Medium (ECM) (ScienCell, Carlsbad, CA, USA) containing 5% FBS, 1% penicillin–streptomycin, and 1% endothelial cell growth supplement; the cells were purchased from ScienCell (Catalog #8000, Carlsbad, CA, USA). All HUVECs used in this study were between passages P2 and P4.

### 4.2. Flow Cytometric Characterization

Sample preparation began with detachment in 0.25% trypsin-EDTA (Gibco, Grand Island, NY, USA) and suspension in phosphate-buffered saline (PBS) (Servicebio, Wuhan, China) containing 0.1% bovine serum albumin (BSA) (Solarbio, Beijing, China). For antibody labeling, the cells were kept in the dark at 4 °C for 30 min with anti-CD34, anti-CD29, anti-CD73, anti-CD105, anti-CD45, and anti-CD90 antibodies (BioLegend, San Diego, CA, USA), using an antibody isotype control. P3 hPDLSCs were first screened on the NovoCyte flow cytometer (Agilent Technologies, San Diego, CA, USA); the resulting data were then analyzed in NovoExpress software (v1.5.0).

### 4.3. Multipotent Differentiation Potential of hPDLSCs

hPDLSCs were examined for multipotent differentiation potential by directing the cells toward osteogenic and adipogenic lineages. At 80% confluence, hPDLSCs were then cultured in osteogenic medium (OM) (PD-014, Procell, Wuhan, China). The osteogenic readout comprised ALP staining on day 7, followed, on day 21, by ARS staining to visualize calcium nodules. The wells were rinsed and imaged by inverted optical microscopy (Olympus, Tokyo, Japan).

To facilitate adipogenesis, hPDLSCs were cultured to attain 80% confluence. Subsequently, the cells were exposed to adipogenic medium (ProCell, Wuhan, China) for 3 weeks. After fixing, lipid droplets were visualized after staining the cells with ORO (Solarbio, Beijing, China).

### 4.4. ALP Staining

For ALP staining after 7 days of culture, hPDLSCs underwent a PBS rinse and fixation at room temperature in 4% paraformaldehyde before 15 min of staining with the BCIP/NBT kit (Solarbio, Beijing, China), after which the cells were examined with a light microscope.

### 4.5. ARS Staining

hPDLSCs were grown for 3 weeks in osteogenic medium and rinsed with PBS. After fixing the cells for 30 min in 4% paraformaldehyde, followed by three washes, 1% ARS (Solarbio, Beijing, China; pH 4.2) was introduced before incubation of the cells at 20–25 °C for 15 min. Following additional washes, inverted light microscopy (Olympus, Tokyo, Japan) was performed for visualizing orange–red mineralized nodules.

### 4.6. Cell Transfection and miR-508-5p Oligonucleotides

miR-508-5p inhibitors, mimics, and corresponding negative control oligonucleotides were chemically synthesized by GenePharma (Shanghai, China). Cell transfection was carried out with 50 nM oligonucleotides for 6 h–12 h using HighGene Plus Transfection Reagent (ABclonal, Wuhan, China) following the manufacturer’s instructions. The results related to the transfection efficiency of miR-508-5p oligonucleotides are provided in Figure S1 of Supplementary Materials. The oligonucleotide sequences are provided in Table 1.

### 4.7. qRT-PCR

Isolation of total RNA followed the manufacturer’s protocol for TRIzol reagent (Invitrogen, Waltham, MA, USA). RNA was reverse-transcribed to generate cDNA. Gene messenger RNA levels were quantified by quantitative polymerase chain reaction (qPCR), with normalization to GAPDH. Relative gene-expression fold changes were calculated with the 2^−ΔΔCt^ approach; Table 2 contains the primer sequences used for each gene.

### 4.8. CCK-8 Assay

Cells were cultivated for various time periods (days 1, 3, 5, and 7) after plating on 96-well plates (cell density: 1.5 × 10^3^ cells/well). CCK-8 (Beyotime Biotechnology, Shanghai, China) provided the cell-viability readout, based on absorbance at 450 nm.

### 4.9. Scratch Wound Healing Assay

Following plating on a 6-well plate, transfected cells were grown to achieve cell confluency of approximately 70–80%. By using a plastic tip, a straight-line scratch was created in the monolayers of cells. Cell debris was discarded by PBS washing. The culture conditions for the scratched cells consisted of serum-free medium. Microscopy images were acquired at 0 h to 24 h following scratch creation.

### 4.10. Transwell Assay

The Transwell procedure began by loading transfected cells in serum-free medium into the upper compartment, while 10% FBS in the lower compartment supplied the chemotactic stimulus. Incubation then continued for 24 h at 37 °C; after this interval, non-migrated cells were cleared from the upper compartment, and cells that had traversed to the lower compartment were stained with 0.1% crystal violet for 10 min before microscopic examination.

### 4.11. Matrigel Angiogenesis Assay

The assay setup used Matrigel-coated 96-well plates (50 μL/well), into which HUVECs in culture medium were introduced at 1 × 10^4^ cells/50 μL. At the 6 h endpoint, an inverted microscope (Olympus, Tokyo, Japan) provided images for subsequent tube-formation quantification in ImageJ software (version 1.54, Bethesda, MD, USA).

### 4.12. CAM Assay

Preparation for the CAM assay proceeded in three stages: fertilized chicken embryos were first kept for 5 days under 38.3 °C and 60% humidity; the eggshell was then opened with a 1 cm circular window and the shell membrane removed to expose the CAM; finally, 200 μL of conditioned medium from the different treatment groups was placed on the CAM. Following the additional 5 days of incubation, CAM specimens were collected and fixed for 20 min in 4% paraformaldehyde. For the vascular readout, a light microscope was used first to examine the vascular structures, after which new blood vessel junctions were quantified with AngioTool software (version 0.6a, Bethesda, MD, USA).

### 4.13. Generation of Stable miR-508-5p Knockdown hPDLSCs Using Lentiviral Vectors

Lentiviral vectors, namely, pLV-hEF1a-EGFP-2A-Puro-8 × Sponge targeting miR-508-5p (LV miR-508-5p) as well as non-targeting control pLV-hEF1a-EGFP-2A-Puro (LV Ctrl), were purchased from SyngenTech (Beijing, China).

After seeding 6-well plates with hPDLSCs (5 × 10^4^ cells/mL), the cells were transferred to a humidified incubator to permit growth. After cell confluence of 40% had been reached, the medium was substituted with a virus-containing medium (multiplicity of infection: 50) containing 8 µg/mL Polybrene. After 12 h–24 h, fresh complete medium was added after the previous medium was removed. Selection with puromycin (2 µg/mL) began 72 h post-infection and continued for 48 h. After fluorescence microscopy confirmed 100% enhanced green fluorescent protein-positive cells, selection and expansion were confirmed by decreasing puromycin concentration to 1 µg/mL. The cells were then harvested, followed by qRT-PCR and Western blot analyses to confirm the efficiency of miR-508-5p knockdown (Supplementary Materials Figure S1). Validated cells were frozen to establish the stable miR-508-5p--silenced hPDLSCs.

### 4.14. In Vivo Experiments

Prior to the experiments, 10 healthy male BALB/c nude (nu/nu) mice (6 weeks of age; Beijing Vital River Laboratory Animal Technology Co., Ltd., Beijing, China) were acclimatized for 1 week under standard housing conditions. Mice were housed in a specific pathogen-free (SPF) facility, and all surgical procedures were performed under SPF conditions.

The four experimental groups were as follows: (1) β-TCP group, (2) β-TCP + OM-induced hPDLSC group, (3) β-TCP + OM-induced LV Ctrl hPDLSC group, and (4) β-TCP + OM-induced LV miR-508-5p hPDLSC group. Under this design, hPDLSCs were cultured in the designated media for 7 days. β-TCP particles (50–500 μm) (Bicon, Boston, MA, USA) were added to culture media for incubation at 37 °C for 1 h to facilitate cell adhesion. Subsequently, hPDLSCs in Groups 2, 3, and 4 were resuspended and seeded onto the pretreated β-TCP particles.

**Figure 8:**
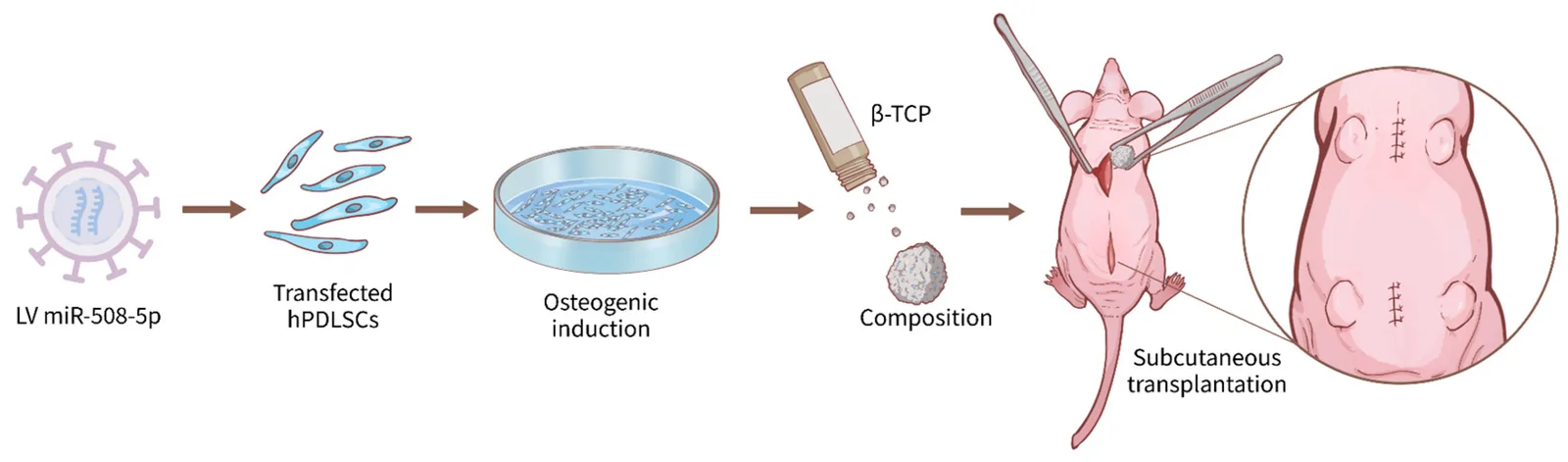
Schematic diagram of the in vivo experimental procedure. Cell–scaffold constructs were prepared by seeding miR-508-5p-silenced hPDLSCs onto β-TCP scaffolds and subsequently implanted subcutaneously into the dorsal region of nude mice. β-TCP, β-tricalcium phosphate.

In in vivo studies, mice were anaesthetized by injecting propofol (26 mg/kg) through the tail-vein. In the dorsal area, 4 enclosed subcutaneous transplantation cavities were created with hemostatic forceps. Mice were randomly assigned to receive four different treatments on four dorsal implantation sites. Each mouse contained one implantation site for each experimental group, thereby minimizing inter-animal variation. β-TCP particles of each group were randomly transplanted into these cavities (5 mice per group per time point) [39]. Following the surgical procedure (Figure 8), the animals were observed two times per day. Predefined humane endpoints were established, including severe body weight loss (>20%), inability to access food or water, severe wound infection that could not be resolved, and other severe conditions affecting animal welfare. However, no anesthesia-related mortality, surgical complications, or incomplete experimental samples occurred, and all animals were included in the subsequent analysis. Mice were then sacrificed through cervical dislocation at 4 weeks and 8 weeks. Tissue collection from all groups was followed immediately by fixation in 4% paraformaldehyde. All surgeries were performed by one investigator, and samples were coded for blinded analysis by a second investigator. The Animal Care and Ethics Committee of Peking University Health Science Center approved all animal studies (PUIRB-LA2024092).

### 4.15. Histological Analysis

The tissues harvested from the dorsal subcutaneous sites of nude mice were fixed and decalcified for 2–4 weeks. Subsequently, 5 μm-thick paraffin sections were prepared and subjected to HE staining (G1005-100ML, Servicebio, Wuhan, China), Masson’s trichrome staining (G1006-100ML, Servicebio, Wuhan, China), COL1A1 immunofluorescence staining (GB155707-100, Servicebio, Wuhan, China), BMP2 immunohistochemical staining (GB15252-100, Servicebio, Wuhan, China), and CD31 immunohistochemical staining (GB11063-2-100, Servicebio, Wuhan, China).

### 4.16. Dual-Luciferase Reporter Assay

The reporter vector was synthesized by GenePharma (Gemma Biotechnology, Shanghai, China). Either wild-type *GLUT1* or its mutant fragment, termed *GLUT1* 3′ untranslated region (3′-UTR)-wt and *GLUT1* 3′-UTR-mut, respectively, was inserted into the vectors. The sequences of the miR-508-5p binding site and the mutant site are as follows: *GLUT1* 3′-UTR-wt (5′-GACCAGUUGGGAGCACUGGAGUG-3′) and *GLUT1* 3′-UTR-mut (5′-GACCAGUUGGGAGCAGACCUCA -3′). To determine whether miR-508-5p binds to *GLUT1* 3′-UTR, 293T cells were seeded in 96-well plates for dual-luciferase reporter assays. The cells were co-transfected with either the *GLUT1* 3′-UTR-wt or the *GLUT1* 3′-UTR-mut reporter plasmid along with mimic NC or miR-508-5p mimics using GP-transfect-Mate (Gemma Biotechnology, Shanghai, China) according to the manufacturer’s instructions. After 48 h, luciferase activity was measured using a Dual-luciferase Reporter Assay Kit (Gemma Biotechnology, Shanghai, China) according to the manufacturer’s instructions. The results of the dual-luciferase reporter assay are provided in Figure S2 of Supplementary Materials.

### 4.17. Statistical Analysis

Data presentation and analysis followed a unified statistical framework. All analyses were performed using SPSS software (version 27.0) (IBM, Armonk, NY, USA). Before statistical analysis, data distribution was assessed using the Shapiro–Wilk test for normality, and homogeneity of variance was evaluated using Levene’s test. For comparisons between two groups, a two-tailed *t*-test was applied to normally distributed data with equal variances, whereas the non-parametric Mann–Whitney U test was used when the data did not satisfy the assumption of normality. For comparisons among multiple groups, one-way analysis of variance (ANOVA) followed by Bonferroni post hoc tests was used when the assumptions for parametric analysis were satisfied. For the in vitro analyses, all statistical tests drew on data from n = 3 independent experiments, each using an independent cell batch. For the in vivo experiments, because each mouse received all four treatments at separate dorsal implantation sites, the four treatment measurements within each mouse were considered correlated observations. Accordingly, one-way repeated-measures analysis of variance was used to account for the within-mouse dependency, followed by Bonferroni-adjusted pairwise comparisons. Statistical significance was defined by *p* < 0.05.

## Figures and Tables

**Table 1:** Sequences of RNA oligos.

RNA Oligo	Sense Strand (5′ to 3′)	Antisense Strand (5′ to 3′)
inhibitor NC	CAGUACUUUUGUGUAGUACAA	-
inhibitor	CAUGAGUGACGCCCUCUGGAGUA	-
mimic NC	UUCUCCGAACGUGUCACGUTT	ACGUGACACGUUCGGAGAATT
mimics	UACUCCAGAGGGCGUCACUCAUG	UGAGUGACGCCCUCUGGAGUAUU

Inhibitor NC, miR-508-5p inhibitor negative control; mimic NC, miR-508-5p mimic negative control.

**Table 2:** Sequences of primers used for qRT-PCR.

Gene	Primer Sequences (5′ to 3′)
*miR-508-5p*	Forward: AACCGCTACTCCAGAGGGC Reverse: CAGAGCAGGGTCCGAGGTA
*U6*	Forward: CGCTTCGGCAGCACATATACTA Reverse: CGCTTCACGAATTTGCGTGTCA
*GAPDH*	Forward: TCATTTCCTGGTATGACAACGA Reverse: GTCTTACTCCTTGGAGGCC
*COL1A1*	Forward: GAGGGCCAAGACGAAGACATC Reverse: CAGATCACGTCATCGCACAAC
*BMP2*	Forward: GACCAAATTATGGAGGAGATG Reverse: AAACAAGTTGTAGCCTTTGG
*OCN*	Forward: GGCGCTACCTGTATCAATGG Reverse: GTGGTCAGCCAACTCGTCA

*GAPDH*, glyceraldehyde-3-phosphate dehydrogenase; *COL1A1*, alpha-1 type 1 collagen; *BMP2*, bone morphogenetic protein 2; *OCN*, osteocalcin.

## Data Availability

The original contributions presented in this study are included in the article/Supplementary Material. Further inquiries can be directed to the corresponding authors.

## Supplementary Materials

The following supporting information can be downloaded at: https://www.mdpi.com/article/10.3390/ijms27188390/s1.

## Abbreviations

ALP	alkaline phosphatase
ARS	alizarin red S
BMP2	Bone morphogenetic protein 2
β-TCP	β-tricalcium phosphate
CAM	chicken embryo chorioallantoic membrane
CM	conditioned medium
COL1A1	Collagen Type I Alpha 1 Chain
HUVECs	human umbilical vein endothelial cells
hPDLSCs	human periodontal ligament stem cells
inhibitor NC	miR-508-5p inhibitor negative control
mimic NC	miR-508-5p mimic negative control
miRNAs	microRNAs
OCN	osteocalcin
ORO	Oil Red O
PDL	periodontal ligament
