# Supplementary material for: Inhibition of miR-508-5p Promotes Osteogenic–Angiogenic Coupling to Enhance Bone Formation
Source: Int J Mol Sci. 2026 Sep 20;27(18):8390. doi: 10.3390/ijms27188390 (PMC13607284; doi:10.3390/ijms27188390)
Supplement: Supplementary file 1 [file ijms-27-08390-s001.zip › ijms-4538724-supplementary.pdf]

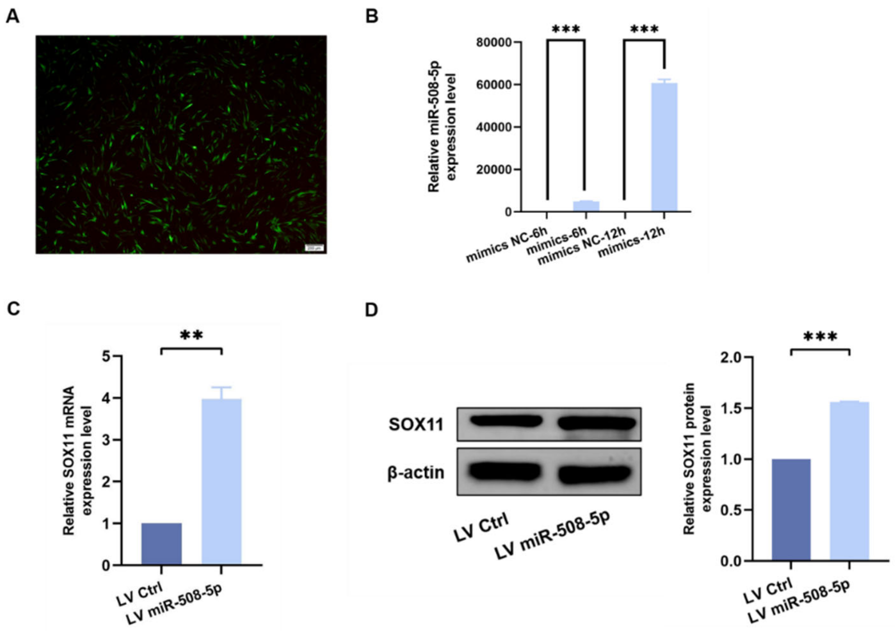

Supplementary Figure S1. Validation of miR-508-5p-related RNA oligonucleotide transfection and lentiviral sponge efficacy. (A) Representative fluorescence microscopy images of cells transfected with carboxyfluorescein (FAM)-labeled miR-508-5p inhibitor (scale bar: 200 μm). (B) miR-508-5p expression levels following transfection with miR-508-5p mimics, as determined by quantitative real-time polymerase chain reaction (qRT-PCR). (C) The mRNA expression level of the downstream target gene SOX11 was determined by qRT-PCR to assess the functional efficacy of the miR-508-5p lentiviral sponge. (D) The protein expression level of SOX11 was determined by Western blotting, with corresponding quantitative analysis, to further assess the functional efficacy of the miR-508-5p lentiviral sponge. $^{**}p < 0.01$, $^{***}p < 0.001$. Note: SOX11, SRY-Box Transcription Factor 11.

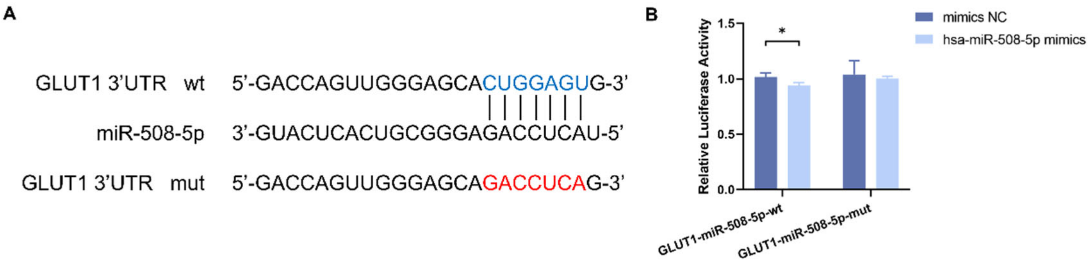

Supplementary Figure S2. Targeting relationship between miR-508-5p and GLUT1. (A) Schematic illustration of the complementary sequence between miR-508-5p and the GLUT1 3′-UTR. Mutation sites on the 3′-UTR of GLUT are highlighted in red. (B) Luciferase reporter assay showing decreased luciferase activity in cells co-transfected with miR-508-5p and wild-type GLUT1 3′-UTR, but not with the mutated 3′-UTR. $^{*}p < 0.05$. Note: GLUT1, glucose transporter type 1; wt, wild-type; mut, mutant.
